# Large-scale comparative genomics and structure–function analysis enables characterization of known and novel genetic determinants of antimicrobial resistance in bacterial pathogens

**DOI:** 10.3389/fmicb.2026.1842956

**Published:** 2026-06-16

**Authors:** Anthony Mannion, Daniel Hooks, Arianna Comendul, Rebecca Spirgel

**Affiliations:** MIT Lincoln Laboratory, Lexington, MA, United States

**Keywords:** antimicrobial resistance, comparative genomic analysis, ESKAPE and enteric pathogens, multidrug resistance mechanisms, protein domain analysis, protein–ligand interaction, structure–function analysis, whole genome sequencing

## Abstract

**Introduction:**

Antibiotics are crucial for preventing infection-induced complications, but their widespread overuse has spurred the evolution of antimicrobial resistance (AMR) mechanisms in pathogens. Data-driven biosurveillance approaches utilizing whole genome sequencing data and computational approaches have the potential to improve the detection and characterization of known and emerging AMR profiles, especially in high-priority ESKAPE, enteric, and sexually-transmitted pathogens.

**Methods:**

In this study, a large-scale analysis of over 70,000 genomes representing 39 pathogen-antibiotic combinations was performed to identify resistance determinants statistically enriched in antibiotic resistant strains.

**Results:**

Using a kmer-based GWAS approach, over 7,000 unique sequences were identified among all resistant genomes. Of these, 1,925 sequences were homologous to known AMR genes, while over 5,000 sequences lacked homology, suggesting novel AMR-associated genes. In addition to identifying the predominant AMR genes for specific pathogen-antibiotic combinations, the findings for this study suggest that horizontal gene transfer mechanisms may influence AMR gene profiles between phylogenetically similar pathogens and antibiotic classes. Likewise, significant associations in co-harbored, multi-drug resistance mechanisms were identified in select pathogens. Protein domains analysis frequently detected efflux/membrane structure and antibiotic-associated metabolism domains in novel AMR-associated proteins, suggesting additional mechanisms potentiate resistance phenotypes. Furthermore, a Random Forest classifier using protein structure, molecular features, and binding affinity profiles to predict protein-antibiotic interactions was developed, identifying several novel proteins that may interact with antibiotics.

**Discussion:**

This study demonstrates the potential of large-scale comparative genomics coupled with AI/ML-based modeling to advance the understanding of AMR threats, thereby enhancing biosurveillance efforts and promoting new strategies to counteract emerging pathogens.

## Introduction

Antibiotics are essential treatments in modern medicine due to their proven efficacy to reduce infection-induced complications from injury or other common procedures like surgery. Consequently, antibiotics are often administered prophylactically and frequently without prior diagnostic tests to confirm pathogen target susceptibility. Wide-spread overuse of antibiotics over the last century has enabled pathogens to acquire/evolve antimicrobial resistance (AMR) mechanisms to counteract these treatments. Currently, AMR pathogens are attributed to greater than 2.8 million infections and cause 35,000 deaths per year in the United States (Flynn and Guarner, 2023). Globally, over 5 million deaths annually are due to AMR infections (Flynn and Guarner, 2023; Ho et al., 2025). By 2050, AMR pathogens are predicted to cause greater than 10 million deaths per year, which will exceed those caused by cancer (Ho et al., 2025) Thus, AMR is a significant threat jeopardizing the treatment of pathogenic infections.

Over the last several decades, numerous resistance mechanisms have been identified in pathogens that drive AMR phenotypes (Darby et al., 2023). Identification of these mechanisms has relied on laboratory-based assays for culture/isolation of suspect resistant strains and screening for antibiotic susceptibility, followed by characterization and experimental confirmation of the genetic determinants (Hassall et al., 2024). Thus, current approaches for AMR identification are labor-, cost-, and time-intensive, which pose significant challenges for understanding the epidemiology of AMR pathogens (Hassall et al., 2024). As a result of these research and knowledge gaps, understanding the prevalence and distribution of AMR genes, especially in high-priority pathogens such as ESKAPE+ (e.g., *Enterococcus faecium*, *Staphylococcus aureus*, *Klebsiella pneumoniae*, *Acinetobacter baumannii*, *Pseudomonas aeruginosa*, *Enterobacter species*, *Escherichia coli*), enteric and sexually-transmitted infections, has been hindered (Miller and Arias, 2024). In turn, these limitations have hampered the development of improved strategies to detect, prevent, and treat known and emerging AMR pathogens, such as novel diagnostic or treatment modalities.

Since the advent of next-generation sequence technologies, public databases that incorporate whole genome sequencing (WGS) with laboratory-based antibiograms have been developed for pathogenic strains and offer the potential to identify reservoirs of genes associated with AMR (Waddington et al., 2022). By utilizing large open-source datasets, it may be feasible to define with statistical confidence which genetic mechanisms predominant for AMR pathogens using comparative genomic analyses akin to genome-wide association studies (GWAS) (Mosquera-Rendón et al., 2023). Genomics-based profiling of AMR patterns could therefore augment empirical decision-making algorithms for antimicrobial therapies when infections are suspected in patients (Sherry et al., 2025). Furthermore, recent advances in bioinformatic and computational biology capabilities via artificial intelligence (AI) and machine learning (ML) tools, such as 3D protein structure prediction and ligand docking modeling (Sim et al., 2025). provide opportunities to simulate and infer biological outcomes. These structure–function modeling approaches have the potential to screen and detect protein–ligand interactions at high-throughput scales, which otherwise may not be feasible by traditional laboratory experiments (Pei, 2024). When applied to microbial pathogens, structure–function modeling tools could facilitate the identification and characterization of mechanisms that promote AMR, including potentially novel genetic determinants. Thus, the hypothesis of this study is that large-scale comparative genomic coupled with AI/ML-based structure–function modeling can be leveraged to enhance the identification and characterization of known and novel AMR mechanism in pathogens.

**Figure 1:**
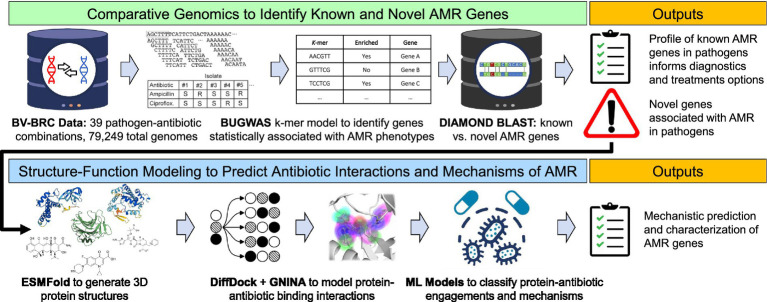
Overview of study. Comparative genomics was performed using paired genome and antibiogram data from the BV-BRC database, and BUGWAS was used to identify *k*-mer sequences statistically associated with antimicrobial resistance (AMR). DIAMOND BLAST was then performed to differentiate known from putative novel AMR genes. EMSFold, followed by DiffDock and GNINA, was used for structure–function modeling of protein-antibiotic interactions. A novel machine learning model was developed to further classify protein-antibiotic interactions associated with enzyme-mediated inactivation mechanisms.

To this end, over 70,000 genomes, representing 39 unique pathogen-antibiotic combinations, were analyzed for AMR-associated proteins using a kmer-based profiling approach, followed by AI/ML-based modeling to characterize the mechanism of interaction between AMR proteins and the antibiotics molecules (Figure 1). In addition to profiling the frequency of known AMR gene determinants, over 5,000 gene sequences were identified that were statistically associated with drug-resistant phenotypes and lack sequence homology to known AMR genes. A novel ML classification algorithm was developed using structure–function modeling tools to predict interactions between AMR genes and antibiotic targets. The findings from this study provide new perspectives on the prevalence and distribution of known AMR gene variants and facilitate the mechanistic characterization of novel protein sequences associated with AMR using emerging AI/ML modeling approaches.

## Methods

### Open-source data and comparative genomics

The API for Bacterial and Viral Bioinformatics Resource Center (BV-BRC) (Olson et al., 2023) was utilized to download annotated proteins from WGS for taxa with laboratory-based antibiograms present in the metadata (accessed March 25, 2025). Antibiotic susceptibility and resistance labels for genome were defined on the antibiogram metadata provided for the strain. Up to 1,500 random genomes for resistant and susceptible strains per pathogen-antibiotic combination were selected for analysis by DBGWAS (De Bruijn Graph Genome-wide association study), under default settings to identify kmers associated with AMR (Supplementary Table 1) (Jaillard et al., 2018). For DBGWAS analysis, an Intel Xeon Platinum 8,260 Processor with 375 GB of RAM was used. Kmers enriched in resistant genomes with a *Q*-value of ≤1E−10 were then mapped to annotated protein sequences. DIAMOND (double index alignment of next-generation sequencing data) was performed against the Comprehensive Antibiotic Resistance Database (CARD, version 4.0.0) to identify homologs to known AMR genes and differentiate non-known AMR genes (Alcock et al., 2023; Buchfink et al., 2021). Identical sequences for non-known AMR genes were de-duplicated to generate representative gene sets. InterProScan (version 5.64-96.0) was used to annotate protein domains for gene sequences (Jones et al., 2014). De-duplicated AMR genes were also clustered into representative gene sequenced using DIAMOND based on a percent identity of 30%. Code to reproduce the comparative genomics analysis is available at https://github.com/mit-ll/AMR_2025.

### 3D protein structure and ligand docking

ESM-Fold (version 2) was used to generate 3D protein structure (Lin et al., 2023). DiffDock2 was then used to dock a seed ligand (e.g., isoniazid) in 3D protein structure to identify the generalized ligand binding region (Corso et al., 2024). Next, GNINA (version 1.1) under the vinardo scoring function was used to refine the ligand pose within the binding box and calculate the predicted binding affinity (McNutt et al., 2021). For DiffDock and GNINA, up to two Nvidia Volta V100 GPUs were used. Code to reproduce the 3D protein structure and ligand docking modeling is available at https://github.com/mit-ll/AMR_2025.

### ML models for classification of protein–ligand interactions

Data was collected from the CARD database consisting of 6,300 proteins and 641 ligands, resulting 4,038,300 protein–ligand combinations. For every combination that had a known interaction, it was labeled as 1. For all other combinations, those without a known interaction, a label of 0 was applied. Only proteins that confer resistance as inactivation enzymes to beta-lactam, aminoglycoside, tetracycline, or macrolides were used for training and testing due to class imbalances for other mechanism of action types and molecule types. Randomly selected subsets of proteins and ligands were used for the test set. Any combination that included a protein or a ligand in the test set was excluded from training.

The set of ligands (*L*) and proteins (*P*) was defined as:

$$L=(l_{1},l_{2},\ldots ,l_{n}),\text{where}\;n=|L|$$

$$P=(p_{1},p_{2},\ldots ,p_{m}),\text{where}\;m=|P|.$$

L and P were randomly sampled to create subsets:

$$L^{\prime }\subseteq L,P^{\prime }\subseteq P.$$

The training set was created by:

$$[(L-L^{\prime })\times (P-P^{\prime })]-(L,P\in [\begin{array}{l} d(l,l_{i})\times b(p,p_{i}),l_{i}\\ \times p_{i}=1,l\times p\neq 1 \end{array}]),$$

where d(x,y) is the Tanimoto similarity of l and $l_{i}$ that is greater than a threshold t , and b(x,y) is the sequence similarity (based on DIAMOND analysis) between protein p and $p_{i}$ >30%.

The test set is the cross of the randomly sampled ligand and protein:

$$L^{\prime }\times P^{\prime }.$$

ESM-Fold was used to represent the proteins as embedded vectors. After determining ligand docking sites with DiffDock2, GNINA was used to calculate the affinity (kcal/mol), root mean squared error, convolutional neural network score, convolutional neural network affinity, and convolutional neural network variance. For each ligand, using their SMILES string representation, RDKit was used to calculate circular fingerprints, MACCSkey fingerprint, Avalon fingerprint, ERG fingerprint, and rdkit features. Protein embedding, affinity output, and molecular features for each combination were used as features and the label of known or unknown interaction as labels. Using the training set, Random Forest and XGboost classifier models were trained. The probability thresholds for each classifier model were assessed to optimize sensitivity versus precision such that the known combinations were correctly classified without overestimating the unknown. A probability threshold of 0.5 was selected for the Random Forest model and 0.25 for the XGboost model to balance tradeoffs between sensitivity and precision for identification of known versus unknown protein–ligand interactions (Supplementary Figure 1). At these thresholds, performance metrics (precision, sensitivity, specificity, AUC, accuracy, PRAUC) were compared between the models across the average of all interaction pairs and per antibiotic class. For training and testing ML classifiers, Intel Xeon E5-2683 v3 Processors with 28 cores and 256 GB of RAM were used. Code to reproduce the ML model development and analysis is available at https://github.com/mit-ll/AMR_2025.

### Statistical analysis

Chi-square tests were performed to analyze the statistical co-association between pairs of on-target AMR genes in pathogen-antibiotic combinations. Likewise, statistical co-association between on- and off-target AMR gene pairs was determined using chi-square tests as well. Briefly, contingency tables were created to determine the prevalence of genomes with and without AMR genes detected to perform the chi-square test. All gene permutations were tested. False Discovery Rate (FDR) adjustments were performed using the Benjamini–Hochberg method, and an FDR-corrected *p*-value < 0.01 was considered statistically significant. Odds ratios were calculated from the contingency tables to inform if genes were positively or inversely associated. Statistical analysis code is available at https://github.com/mit-ll/AMR_2025.

## Results

### DBGWAS-based identification of AMR-associated AMR genes

136,297 total genomes from nine different genera, representing 11 unique taxonomies, with laboratory-based antibiogram data for 24 different antibiotics were assessed from the BV-BRC database (Supplementary Table 1). In total, 39 unique pathogen-antibiotic combinations were evaluated. Due to computational/memory limitations using DBGWAS, a maximum of 1,500 genomes from resistant and susceptible genomes (*n* = 3,000 total genomes maximum) could be compared per pathogen-antibiotic combination (Supplementary Table 1), which represented 79,249 total genomes.

**Figure 2:**
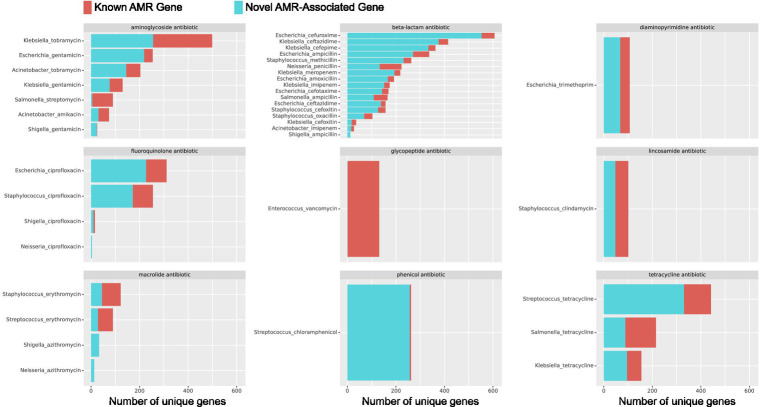
The total number of unique sequences detected for known and novel AMR-associated genes are shown for each pathogen-antibiotic combination, per antibiotic class.

Across the 39 pathogen-antibiotic combinations, 7,149 unique sequences were identified that were enriched in the genomes of resistant strains. Of these sequences, 1,925 (29.9%) unique were homologous to known AMR-associated genes in the CARD, whereas 5,224 (73.1%) lacked homology hits (henceforth referred as “novel” AMR-associated genes). The distribution of known versus “novel” AMR-associated genes varied considerably per pathogen-antibiotic combination (Figure 2). Novel AMR-associated genes contributed >50% of total enriched sequences for 31/39 pathogen-antibiotic combinations.

Of the 39 per pathogen-antibiotic combinations, 35 had known AMR-associated genes detected. The four pathogen-antibiotic combinations that lacked homology to known AMR-associated genes were *Shigella*-azithromycin, *Neisseria*-azithromycin, *Shigella*-ampicillin, and *Neisseria*-ciprofloxacin. Two pathogen-antibiotic combinations, *Enterococcus*-vancomycin and *Salmonella*-streptomycin, primarily harbored known AMR genes, with less <5% genomes co-harboring novel AMR-associated sequences. For the remaining 37 pathogen-antibiotic combos, novel AMR-associated sequences were identified in >10% of the genomes analyzed. In general, a higher percentage of genomes per pathogen-antibiotic combination tended to co-harbor both known and novel AMR-associated sequences. There were subsets of genomes that lacked both known and novel AMR-associated genes (Supplementary Figure 2), suggesting the presence of undetected AMR-associated sequences that may have been statistically underpowered for detection using DBGWAS, possibly due low strain abundance in the BV-BRC database.

### On-target AMR gene profiles

**Figure 3:**
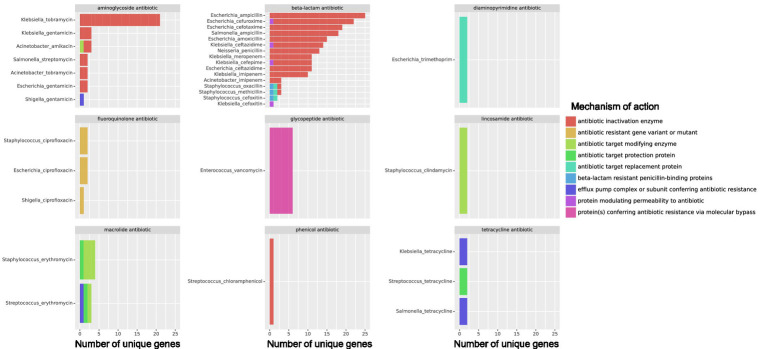
The mechanism of action for the number of unique on-target AMR genes are shown for each pathogen-antibiotic combination, per antibiotic class.

Known AMR genes were then assessed for whether they conferred resistance to the expected antibiotic class in pathogen-antibiotic combinations (i.e., distinguishing on-target from off-target AMR genes). For 35/39 pathogen-antibiotic combos, at least one on-target AMR gene detected. However, most pathogen-antibiotic combos had several on-target AMR genes identified, with up to 25 unique gene sequences for beta-lactam resistance (Figure 3).

To determine if genomes tended to co-harbor specific on-target AMR gene pairs, chi-square tests were performed. Among all pathogen-genome combinations, only 17 on-target AMR gene pairs were found to be significantly associated when analyzing pairs found in >5% of genome (*p*-value < 0.01, odds-ratio >1) (Table 1). The majority of co-harbored AMR genes (10/17 pairs) were found in *Enterococcus* genomes and were due to the gene cluster that confers resistance to vancomycin (Table 1). Interestingly, the remaining co-harbored AMR genes represented the same mechanisms of resistance, except for a single pair found in *Streptococcus*-erythromycin in which the two genes had different mechanisms of action. This observation suggests that it may be uncommon for genomes to harbor multiple different genes targeting the same antibiotic, possibly because some resistance mechanisms introduce redundancy that may reduce the overall fitness of the organism. It should be noted that efflux pumps were not considered in the co-associated gene comparison because these mechanisms potentiate non-specific resistance to broad classes of antibiotics.

**Table 1:** Characteristics of statistically co-associated on-target AMR gene pairs per pathogen-antibiotic combination as determined by chi-square analysis.

Pathogen-antibiotic combination	Gene 1	Gene 1 MOA	Gene 1 antibiotic target	Gene 2	Gene 2 MOA	Gene 2 antibiotic target	% genomes	Odds ratio	FDR-adjusted *p*-value
*Enterococcus*-vancomycin	vanA (ARO:3000010)	Glycopeptide resistance cluster	Glycopeptide	vanS gene in vanA cluster (ARO:3002931)	Glycopeptide resistance cluster	Glycopeptide	82.1	43,848	2.37E−260
*Enterococcus*-vancomycin	vanA (ARO:3000010)	Glycopeptide resistance cluster	Glycopeptide	vanH gene in vanA cluster (ARO:3002942)	Glycopeptide resistance cluster	Glycopeptide	82.5	inf	2.91E−267
*Enterococcus*-vancomycin	vanA (ARO:3000010)	Glycopeptide resistance cluster	Glycopeptide	vanX gene in vanA cluster (ARO:3002949)	Glycopeptide resistance cluster	Glycopeptide	82.5	inf	2.91E−267
*Enterococcus*-vancomycin	vanA (ARO:3000010)	Glycopeptide resistance cluster	Glycopeptide	vanY gene in vanA cluster (ARO:3002955)	Glycopeptide resistance cluster	Glycopeptide	76.2	2,645.299	7.17E−183
*Enterococcus*-vancomycin	vanS gene in vanA cluster (ARO:3002931)	Glycopeptide resistance cluster	Glycopeptide	vanH gene in vanA cluster (ARO:3002942)	Glycopeptide resistance cluster	Glycopeptide	82.1	inf	1.01E−261
*Enterococcus*-vancomycin	vanS gene in vanA cluster (ARO:3002931)	Glycopeptide resistance cluster	Glycopeptide	vanX gene in vanA cluster (ARO:3002949)	Glycopeptide resistance cluster	Glycopeptide	82.1	inf	1.01E−261
*Enterococcus*-vancomycin	vanS gene in vanA cluster (ARO:3002931)	Glycopeptide resistance cluster	Glycopeptide	vanY gene in vanA cluster (ARO:3002955)	Glycopeptide resistance cluster	Glycopeptide	76.1	911.7244	1.18E−182
*Enterococcus*-vancomycin	vanH gene in vanA cluster (ARO:3002942)	Glycopeptide resistance cluster	Glycopeptide	vanX gene in vanA cluster (ARO:3002949)	Glycopeptide resistance cluster	Glycopeptide	82.5	inf	1.87E−268
*Enterococcus*-vancomycin	vanH gene in vanA cluster (ARO:3002942)	Glycopeptide resistance cluster	Glycopeptide	vanY gene in vanA cluster (ARO:3002955)	Glycopeptide resistance cluster	Glycopeptide	76.3	inf	5.07E−184
*Enterococcus*-vancomycin	vanX gene in vanA cluster (ARO:3002949)	Glycopeptide resistance cluster	Glycopeptide	vanY gene in vanA cluster (ARO:3002955)	Glycopeptide resistance cluster	Glycopeptide	76.3	inf	5.07E−184
*Escherichia*-ciprofloxacin	*E. coli* gyrA (ARO:3003294)	Antibiotic resistant DNA topoisomerase subunit	Fluoroquinolone	*E. coli* parC (ARO:3003308)	Antibiotic resistant DNA topoisomerase subunit	Fluoroquinolone	84.7	112.1707	4.01E−69
*Klebsiella*-tobramycin	AAC(6′)-Ib10 (ARO:3002581)	Aminoglycoside modifying enzyme	Aminoglycoside	aadA (ARO:3002601)	Aminoglycoside modifying enzyme	Aminoglycoside	40.1	4.190979	7.90E−21
*Klebsiella*-tobramycin	AAC(6′)-Ib10 (ARO:3002581)	Aminoglycoside modifying enzyme	Aminoglycoside	AAC(3)-IIe (ARO:3004621)	Aminoglycoside modifying enzyme	Aminoglycoside	28.3	2.626489	1.86E−08
*Klebsiella*-tobramycin	aadA (ARO:3002601)	Aminoglycoside modifying enzyme	Aminoglycoside	aadA2 (ARO:3002602)	Aminoglycoside modifying enzyme	Aminoglycoside	27.8	4.559945	3.08E−33
*Salmonella*-streptomycin	APH(3″)-Ib (ARO:3002639)	Aminoglycoside modifying enzyme	Aminoglycoside	APH(6)-Id (ARO:3002660)	Aminoglycoside modifying enzyme	Aminoglycoside	77.4	3,850.733	2.54E−169
*Staphylococcus*-ciprofloxacin	*S. aureus* gyrA (ARO:3003296)	Antibiotic resistant DNA topoisomerase subunit	Fluoroquinolone	*S. aureus* parC (ARO:3003312)	Antibiotic resistant DNA topoisomerase subunit	Fluoroquinolone	90.5	18.55357	1.49E−35
*Streptococcus*-erythromycin	mel (ARO:3000616)	Major facilitator superfamily (MFS) antibiotic efflux pump	Macrolide	msrE (ARO:3003109)	ABC-F ATP-binding cassette ribosomal protection protein	Macrolide	51.9	inf	0

**Figure 4:**
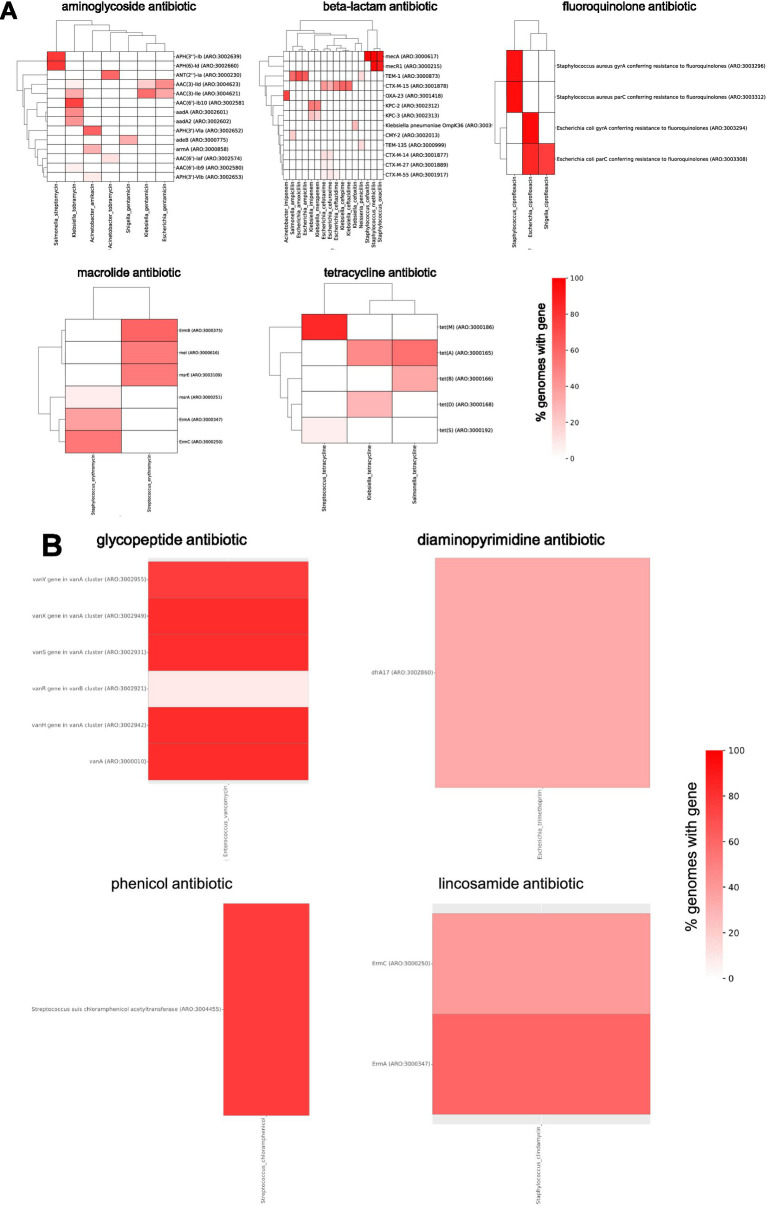
Co-occurrence of known AMR genes across antibiotic classes per pathogen-antibiotic combination was determined by hierarchical clustering based on Euclidean distance of the percentage of genomes containing each gene. **(A)** Clustermaps and **(B)** heatmaps of the abundance of known AMR genes by antibiotic class are shown. Only genes present in ≥5% of genomes were analyzed.

**Figure 5:**
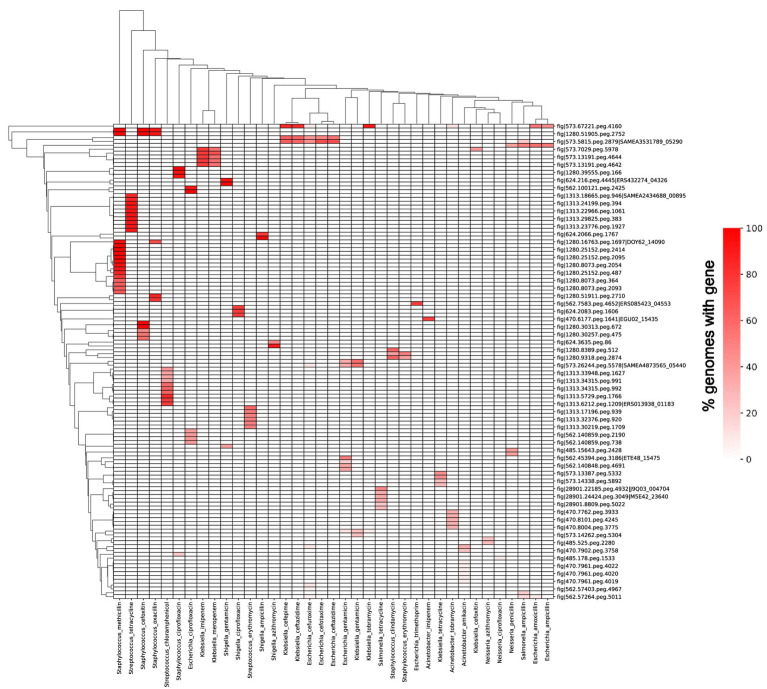
Co-occurrence of novel AMR-associated genes was determined by hierarchical clustering based on Euclidean distance of the percentage of genomes containing each gene. Clustermap of the abundance of novel AMR-associated genes by pathogen-antibiotic combination is shown. Only genes present in ≥5% of genomes were analyzed.

In general, on-target AMR genes tended to converge on a predominate mechanism for resistance based on the antibiotic class analyzed. For example, AMR genes functioning as antibiotic inactivation enzymes were the most commonly occurring mechanisms for beta-lactam and aminoglycoside classes for all pathogen-antibiotic combinations (Figure 3). Likewise, specific AMR genes were predominantly identified for each pathogen-antibiotic combination. Additionally, hierarchal clustering revealed that pathogen-antibiotic combinations with more similar phylogeny and/or antibiotic classes tended to have more related AMR gene profiles (Figure 4). For example, *Escherichia*, *Klebsiella*, and *Salmonella* genomes (all of members of the *Enterobacteriaceae* family) resistant to penicillin-like molecules were enriched in TEM-1 (ARO:3008730). Conversely, *Escherichia* and *Klebsiella* genomes resistant to carbapenem or cephalosporin were enriched in KPC-2 beta-lactamase (ARO:3002312)/KPC-3 (ARO:3002313) or CTX-M-25 (ARO:3001887), respectively. Furthermore, *Acinetobacter* genomes, which are phylogenetically distant from *Enterobacteriaceae* species, that were resistant to carbapenems primarily harbored OXA-23 (ARO:3001418). A similar trend was also observed when novel AMR-associated genes were analyzed by hierarchal clustering in that gene profiles tended to be more similar for pathogen-antibiotic combinations based on phylogeny and/or antibiotic class similarities (Figure 5; Supplementary Figure 3).

### Off-target AMR gene profiles

In addition to known AMR genes conferring resistance to the target antibiotic, off-target AMR genes were identified in 23/39 pathogen-antibiotic combinations, suggesting the potential for multi-drug resistance phenotypes. Off-target AMR genes functioning as efflux pumps or modulators of drug permeability were the most often identified among different pathogen-antibiotic combinations (Supplementary Figure 4). Chi-square tests of the co-association between on- and off-target AMR gene identified 32 gene pairs significantly associated when analyzing pairs found in >5% of genome (*p*-value < 0.01, odds-ratio >1), in which efflux pumps were the most commonly co-harbored mechanism of action found in 8/32 gene pairs (Table 2).

**Table 2:** Characteristics of statistically co-associated on- with off-target AMR gene pairs per pathogen-antibiotic combination as determined by chi-square analysis.

Pathogen-Antibiotic Combination	Gene 1	Gene 1 MOA	Gene 1 antibiotic target	Gene 2	Gene 2 MOA	Gene 2 antibiotic target	% genomes	Odds ratio	FDR-adjusted *p*-value
*Acinetobacter*-amikacin	armA (ARO:3000858)	rRNA methyltransferase conferring antibiotic resistance	Aminoglycoside	mphE (ARO:3003741)	Macrolide inactivation enzyme	Macrolide antibiotic	30.5	inf	3.62E−93
*Enterococcus*-vancomycin	vanA (ARO:3000010)	Glycopeptide resistance cluster	Glycopeptide	vanR gene in vanA cluster (ARO:3002919)	Glycopeptide resistance cluster	Peptide antibiotic	82.1	43,848	2.37E−260
*Enterococcus*-vancomycin	vanS gene in vanA cluster (ARO:3002931)	Glycopeptide resistance cluster	Glycopeptide	vanA (ARO:3000010)	Glycopeptide resistance cluster	Cycloserine-like antibiotic	82.1	43,848	2.37E−260
*Enterococcus*-vancomycin	vanH gene in vanA cluster (ARO:3002942)	Glycopeptide resistance cluster	Glycopeptide	vanA (ARO:3000010)	Glycopeptide resistance cluster	Cycloserine-like antibiotic	82.5	inf	2.91E−267
*Enterococcus*-vancomycin	vanX gene in vanA cluster (ARO:3002949)	Glycopeptide resistance cluster	Glycopeptide	vanA (ARO:3000010)	Glycopeptide resistance cluster	Cycloserine-like antibiotic	82.5	inf	2.91E−267
*Enterococcus*-vancomycin	vanY gene in vanA cluster (ARO:3002955)	Glycopeptide resistance cluster	Glycopeptide	vanA (ARO:3000010)	Glycopeptide resistance cluster	Cycloserine-like antibiotic	76.2	2,645.299	7.17E−183
*Enterococcus*-vancomycin	vanS gene in vanA cluster (ARO:3002931)	Glycopeptide resistance cluster	Glycopeptide	vanR gene in vanA cluster (ARO:3002919)	Glycopeptide resistance cluster	Peptide antibiotic	82.1	inf	1.87E−268
*Enterococcus*-vancomycin	vanH gene in vanA cluster (ARO:3002942)	Glycopeptide resistance cluster	Glycopeptide	vanR gene in vanA cluster (ARO:3002919)	Glycopeptide resistance cluster	Peptide antibiotic	82.1	inf	1.01E−261
*Enterococcus*-vancomycin	vanX gene in vanA cluster (ARO:3002949)	Glycopeptide resistance cluster	Glycopeptide	vanR gene in vanA cluster (ARO:3002919)	Glycopeptide resistance cluster	Peptide antibiotic	82.1	inf	1.01E−261
*Enterococcus*-vancomycin	vanY gene in vanA cluster (ARO:3002955)	Glycopeptide resistance cluster	Glycopeptide	vanR gene in vanA cluster (ARO:3002919)	Glycopeptide resistance cluster	Peptide	76.1	911.7244	1.18E−182
*Escherichia*-cefuroxime	CTX-M-15 (ARO:3001878)	Beta-lactamase	Beta-lactam	catB3 (ARO:3002676)	Chloramphenicol acetyltransferase (CAT)	Phenicol	15.0	137.1058	1.24E−50
*Escherichia*-cefuroxime	CTX-M-15 (ARO:3001878)	Beta-lactamase	Beta-lactam	catB3 (ARO:3002676)	Chloramphenicol acetyltransferase (CAT)	Streptogramin	15.0	137.1058	1.24E−50
*Escherichia*-ciprofloxacin	*E. coli* gyrA (ARO:3003294)	Antibiotic resistant DNA topoisomerase Subunit	Fluoroquinolone	*E. coli* parC (ARO:3003308)	Antibiotic resistant DNA topoisomerase subunit	Disinfecting agents and antiseptics	84.7	112.1707	4.01E-69
*Escherichia*-ciprofloxacin	*E. coli* parC (ARO:3003308)	Antibiotic resistant DNA topoisomerase subunit	Fluoroquinolone	*E. coli* gyrA (ARO:3003294)	Antibiotic resistant DNA topoisomerase subunit	Disinfecting agents and antiseptics	84.7	112.1707	4.01E−69
*Escherichia*-gentamicin	AAC(3)-IIe (ARO:3004621)	Aminoglycoside modifying enzyme	Aminoglycoside antibiotic	tmrB (ARO:3003059)	Tunicamycin resistance protein	Nucleoside antibiotic	34.3	150.8684	1.37E−47
*Escherichia*-trimethoprim	dfrA17 (ARO:3002860)	Antibiotic resistant dihydrofolate reductase	Diaminopyrimidine	qacEdelta1 (ARO:3005010)	Small multidrug resistance (SMR) antibiotic efflux pump	Macrolide antibiotic	30.3	21.10884	5.07E−32
*Escherichia*-trimethoprim	dfrA17 (ARO:3002860)	Antibiotic resistant dihydrofolate reductase	Diaminopyrimidine	qacEdelta1 (ARO:3005010)	Small multidrug resistance (SMR) antibiotic efflux pump	Aminocoumarin antibiotic	30.3	21.10884	5.07E−32
*Escherichia*-trimethoprim	dfrA17 (ARO:3002860)	Antibiotic resistant dihydrofolate reductase	Diaminopyrimidine	qacEdelta1 (ARO:3005010)	Small multidrug resistance (SMR) antibiotic efflux pump	Aminoglycoside antibiotic	30.3	21.10884	5.07E−32
*Escherichia*-trimethoprim	dfrA17 (ARO:3002860)	Antibiotic resistant dihydrofolate reductase	Diaminopyrimidine	qacEdelta1 (ARO:3005010)	Small multidrug resistance (SMR) antibiotic efflux pump	Disinfecting agents and antiseptics	30.3	21.10884	5.07E−32
*Escherichia*-trimethoprim	dfrA17 (ARO:3002860)	Antibiotic resistant dihydrofolate reductase	Diaminopyrimidine	aadA5 (ARO:3002605)	Aminoglycoside modifying enzyme	Aminoglycoside antibiotic	32.6	2,317.714	2.08E−94
*Escherichia*-trimethoprim	sul1 (ARO:3000410)	Sulfonamide resistant sul	Sulfonamide antibiotic	dfrA17 (ARO:3002860)	Antibiotic resistant dihydrofolate reductase	Diaminopyrimidine antibiotic	30.1	19.56571	2.04E−31
*Escherichia*-trimethoprim	sul1 (ARO:3000410)	Sulfonamide resistant sul	Sulfone antibiotic	dfrA17 (ARO:3002860)	Antibiotic resistant dihydrofolate reductase	Diaminopyrimidine antibiotic	30.1	19.56571	2.04E−31
*Klebsiella*-gentamicin	AAC(3)-IIe (ARO:3004621)	Aminoglycoside modifying enzyme	Aminoglycoside antibiotic	tmrB (ARO:3003059)	Tunicamycin resistance protein	Nucleoside antibiotic	33.3	2.025131	9.74E−11
*Klebsiella*-gentamicin	AAC(3)-IId (ARO:3004623)	Aminoglycoside modifying enzyme	Aminoglycoside antibiotic	tmrB (ARO:3003059)	Tunicamycin resistance protein	Nucleoside antibiotic	18.7	22.88775	2.81E−60
*Klebsiella*-tetracycline	tet(A) (ARO:3000165)	Major facilitator superfamily (MFS) antibiotic efflux pump	Tetracycline antibiotic	tetR (ARO:3003479)	Mutant efflux regulatory protein conferring antibiotic resistance	–	45.8	inf	2.46E−73
*Klebsiella*-tetracycline	tet(D) (ARO:3000168)	Major facilitator superfamily (MFS) antibiotic efflux pump	Tetracycline antibiotic	tetR (ARO:3003479)	Mutant efflux regulatory protein conferring antibiotic resistance	–	28.1	30.16274	5.82E−31
*Klebsiella*-tobramycin	aadA (ARO:3002601)	Aminoglycoside modifying enzyme	Aminoglycoside antibiotic	AAC(6′)-Ib10 (ARO:3002581)	Aminoglycoside modifying enzyme	Fluoroquinolone antibiotic	40.1	4.190979	7.90E−21
*Klebsiella*-tobramycin	AAC(6′)-Ib10 (ARO:3002581)	Aminoglycoside modifying enzyme	Aminoglycoside antibiotic	catB3 (ARO:3002676)	Chloramphenicol acetyltransferase (CAT)	Phenicol antibiotic	38.4	7.101639	1.97E−30
*Klebsiella*-tobramycin	AAC(6′)-Ib10 (ARO:3002581)	Aminoglycoside modifying enzyme	Aminoglycoside antibiotic	catB3 (ARO:3002676)	Chloramphenicol acetyltransferase (CAT)	Streptogramin antibiotic	38.4	7.101639	1.97E−30
*Klebsiella*-tobramycin	AAC(6′)-Ib10 (ARO:3002581)	Aminoglycoside modifying enzyme	Aminoglycoside antibiotic	tmrB (ARO:3003059)	Tunicamycin resistance protein	Nucleoside antibiotic	29.4	3.80863	3.79E−14
*Klebsiella*-tobramycin	AAC(3)-IIe (ARO:3004621)	Aminoglycoside modifying enzyme	Aminoglycoside antibiotic	AAC(6′)-Ib10 (ARO:3002581)	Aminoglycoside modifying enzyme	Fluoroquinolone antibiotic	28.3	2.626489	1.86E−08
*Klebsiella*-tobramycin	AAC(6′)-Ib10 (ARO:3002581)	Aminoglycoside modifying enzyme	Aminoglycoside antibiotic	OXA-1 (ARO:3001396)	Beta-lactamase	Beta-lactam antibiotic	38.9	8.496912	5.86E−34
*Klebsiella*-tobramycin	AAC(3)-IIe (ARO:3004621)	Aminoglycoside modifying enzyme	Aminoglycoside antibiotic	OXA-1 (ARO:3001396)	Beta-lactamase	Beta-lactam antibiotic	25.9	11.4832	2.07E−69
*Klebsiella*-tobramycin	AAC(3)-IIe (ARO:3004621)	Aminoglycoside modifying enzyme	Aminoglycoside antibiotic	catB3 (ARO:3002676)	Chloramphenicol acetyltransferase (CAT)	Phenicol antibiotic	25.7	10.83711	5.99E−67
*Klebsiella*-tobramycin	AAC(3)-IIe (ARO:3004621)	Aminoglycoside modifying enzyme	Aminoglycoside antibiotic	catB3 (ARO:3002676)	Chloramphenicol acetyltransferase (CAT)	Streptogramin antibiotic	25.7	10.83711	5.99E−67
*Klebsiella*-tobramycin	AAC(3)-IIe (ARO:3004621)	Aminoglycoside modifying enzyme	Aminoglycoside antibiotic	tmrB (ARO:3003059)	Tunicamycin resistance protein	Nucleoside antibiotic	27.8	57.90865	1.48E−146
*Klebsiella*-tobramycin	AAC(3)-IId (ARO:3004623)	Aminoglycoside modifying enzyme	Aminoglycoside antibiotic	tmrB (ARO:3003059)	Tunicamycin resistance protein	Nucleoside antibiotic	6.1	59.77536	2.97E−31
*Salmonella*-ampicillin	CMY-2 (ARO:3002013)	Beta-lactamase	Beta-lactam antibiotic	ykkC (ARO:3003063)	Subunit of efflux pump conferring antibiotic resistance	Aminoglycoside antibiotic	17.3	2,816.929	5.31E−240
*Salmonella*-ampicillin	CMY-2 (ARO:3002013)	Beta-lactamase	Beta-lactam antibiotic	ykkC (ARO:3003063)	Small multidrug resistance (SMR) antibiotic efflux pump	Aminoglycoside antibiotic	17.3	2,816.929	5.31E−240
*Salmonella*-tetracycline	tet(A) (ARO:3000165)	Major facilitator superfamily (MFS) antibiotic efflux pump	Tetracycline antibiotic	tetR (ARO:3003479)	Mutant efflux regulatory protein conferring antibiotic resistance	–	55.6	136.0778	6.41E−49
*Salmonella*-tetracycline	tet(B) (ARO:3000166)	Major facilitator superfamily (MFS) antibiotic efflux pump	Tetracycline antibiotic	tetR (ARO:3003479)	Mutant efflux regulatory protein conferring antibiotic resistance	–	33.6	10.41443	7.86E−16
*Salmonella*-tetracycline	tet(B) (ARO:3000166)	Major facilitator superfamily (MFS) antibiotic efflux pump	Tetracycline antibiotic	adeN (ARO:3000559)	Resistance-nodulation-cell division antibiotic efflux pump	–	12.0	239.2074	6.72E−76
*Staphylococcus*-ciprofloxacin	*S. aureus* gyrA (ARO:3003296)	Antibiotic resistant DNA topoisomerase subunit	Fluoroquinolone antibiotic	*S. aureus* parC (ARO:3003312)	Antibiotic resistant DNA topoisomerase subunit	Disinfecting agents and antiseptics	90.5	18.55357	1.49E−35
*Staphylococcus*-ciprofloxacin	*S. aureus* parC (ARO:3003312)	Antibiotic resistant DNA topoisomerase subunit	Fluoroquinolone antibiotic	*S. aureus* gyrA (ARO:3003296)	Antibiotic resistant DNA topoisomerase subunit	Disinfecting agents and antiseptics	90.5	18.55357	1.49E−35
*Staphylococcus*-clindamycin	ErmA (ARO:3000347)	rRNA methyltransferase conferring antibiotic resistance	Lincosamide antibiotic	ANT(9)-Ia (ARO:3002630)	Aminoglycoside modifying enzyme	Aminoglycoside antibiotic	60.2	inf	1.16E−100
*Staphylococcus*-erythromycin	ErmA (ARO:3000347)	rRNA methyltransferase conferring antibiotic resistance	Macrolide antibiotic	ANT(9)-Ia (ARO:3002630)	Aminoglycoside modifying enzyme	Aminoglycoside antibiotic	37.5	inf	5.55E−198
*Streptococcus*-erythromycin	mel (ARO:3000616)	Major facilitator superfamily (MFS) antibiotic efflux pump	Macrolide antibiotic	msrE (ARO:3003109)	ABC-F ATP-binding cassette ribosomal protection protein	Phosphonic acid antibiotic	51.9	inf	0
*Streptococcus*-erythromycin	mel (ARO:3000616)	Major facilitator superfamily (MFS) antibiotic efflux pump	Macrolide antibiotic	msrE (ARO:3003109)	ABC-F ATP-binding cassette ribosomal protection protein	Streptogramin antibiotic	51.9	inf	0
*Streptococcus*-erythromycin	mel (ARO:3000616)	Major facilitator superfamily (MFS) antibiotic efflux pump	Macrolide antibiotic	msrE (ARO:3003109)	ABC-F ATP-binding cassette ribosomal protection protein	Lincosamide antibiotic	51.9	inf	0
*Streptococcus*-erythromycin	mel (ARO:3000616)	Major facilitator superfamily (MFS) antibiotic efflux pump	Macrolide antibiotic	msrE (ARO:3003109)	ABC-F ATP-binding cassette ribosomal protection protein	Pleuromutilin antibiotic	51.9	inf	0
*Streptococcus*-erythromycin	mel (ARO:3000616)	Major facilitator superfamily (MFS) antibiotic efflux pump	Macrolide antibiotic	msrE (ARO:3003109)	ABC-F ATP-binding cassette ribosomal protection protein	Nucleoside antibiotic	51.9	inf	0
*Streptococcus*-erythromycin	mel (ARO:3000616)	Major facilitator superfamily (MFS) antibiotic efflux pump	Macrolide antibiotic	msrE (ARO:3003109)	ABC-F ATP-binding cassette ribosomal protection protein	Antibiotic without defined classification	51.9	inf	0
*Streptococcus*-erythromycin	msrE (ARO:3003109)	ABC-F ATP-binding cassette ribosomal protection protein	Macrolide antibiotic	mel (ARO:3000616)	Major facilitator superfamily (MFS) antibiotic efflux pump	Tetracycline antibiotic	51.9	inf	0

### Functional domain predictions of novel AMR-associated genes

**Figure 6:**
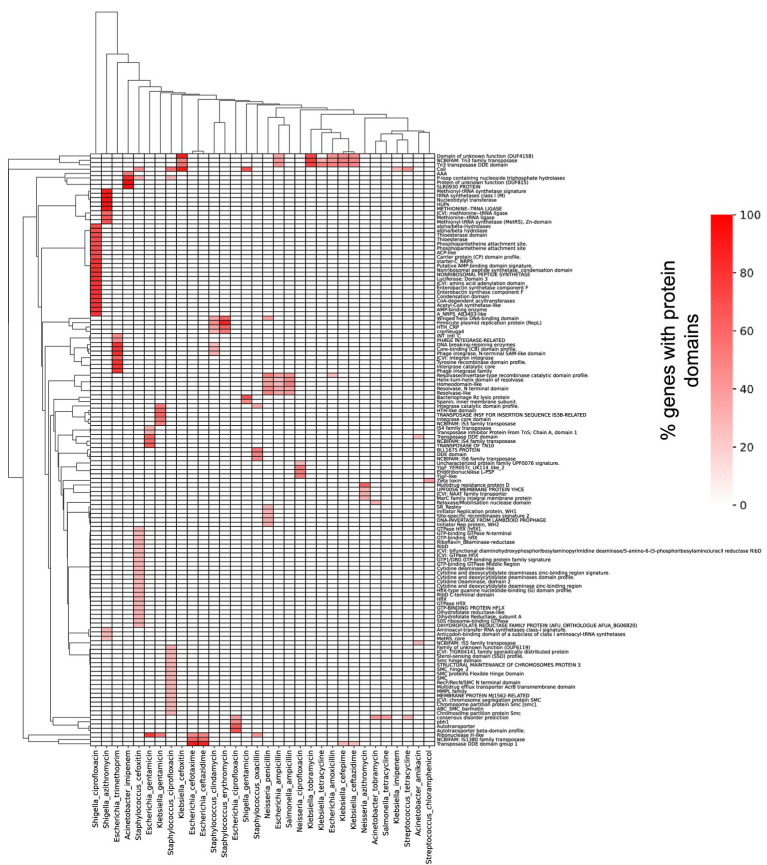
Protein domains were identified in novel AMR-associated genes using InterProScan. Co-occurrence of protein domains in novel AMR-associated genes across pathogen-antibiotic combinations was determined by hierarchical clustering based on Euclidean distance of the percentage of genes with protein domains. Clustermap of the abundance of protein domains per pathogen-antibiotic combination is shown. Only protein domains present in ≥25% of genes for a pathogen-antibiotic combination were analyzed.

To gain the functional insights of “novel” AMR-associated genes, protein domains were predicted using InterProScan. Protein domains were identified in 4,001/5,224 (70.8%) of the novel AMR-associated gene sequence. A total of 901 unique domains were present, representing a wide diversity of potential functions and mechanisms. Hierarchal cluster analysis indicated the types and distribution of proteins domains tended to associate with more similar pathogen-antibiotic combinations, which was also observed for known on- and off-target AMRs genes as described above (Figure 6). Across pathogen-antibiotic combinations, the most commonly observed domains were related to horizonal gene transfer and included plasmid- and phage-mediated genetic exchange mechanisms such as ribonuclease, resolvase, integrase, recombinase, transposase, endonuclease proteins. This finding supports the importance of genetic exchange being a critical mechanism driving the propagation of antibiotic resistance in pathogens. Aside from horizonal gene transfer, domains implicated in molecular transporters and cell wall structure were frequently observed, which may expand the repertoire of efflux pumps/modulators of membrane permeability that contribute to resistance (Supplementary Figures 5 and 6). Interestingly, several domains related to oxidation–reduction mechanisms, such as FAD-linked oxidoreductase, NADP-dependent oxidoreductase, and flavoprotein-like domains were identified (Supplementary Figures 5 and 6). Domains associated with antibiotic/natural product metabolism including nonribosomal peptide synthetase, antibiotic biosynthesis monooxygenase, isochorismatase-like hydrolase, chloramphenicol acetyltransferase-like, dihydroxybiphenyl dioxygenase were also identified (Supplementary Figures 5 and 6). These protein domains may contribute to antibiotic resistance via degradation mechanisms. Notably, several domains similar to known AMR mechanism like dihydrofolate reductase subunits, pentapeptide repeat-like, DNA topoisomerase, tetracycline repressor-like and were detected as well (Supplementary Figures 5 and 6).

### Structure–function modeling of novel AMR-associated genes

**Figure 7:**
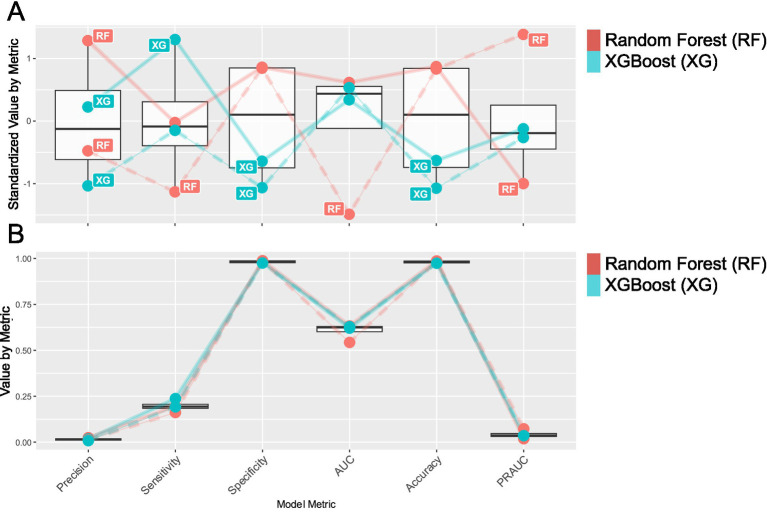
Machine learning classifier models were developed to predict protein-antibiotic interactions based on *in silico* binding affinity, protein sequence embedding, and molecular features. Comparison of standardized **(A)** and non-standardized **(B)** model performance metrics for random forest (RF) and XGBoost (XG) classifiers are shown. Solid lines represent model performance over all values and dashed lines represent average model performance across antibiotic class.

Random Forest and XGboost models were created to classify protein-antibiotic interactions based on predicted protein–ligand binding affinity profiles, protein sequence embeddings and antibiotic molecular fingerprints/descriptors. As shown in the parallel coordinate plots for standardized and non-standardized performance metrics in (Figure 7), the Random Forest model achieved higher precision, specificity, AUC, and accuracy values compared to XGboost. PRAUC scores were comparable between the models, while XGboost achieved higher sensitivity (Figure 7). Both models yielded better performance on macrolides compared to other antibiotic classes (Supplementary Figure 7). Aminoglycosides and beta-lactams had higher number of estimated interactions with lower specificity and accuracy but higher sensitivity.

Additionally, both models were assessed on different data subsets based on whether the proteins and ligands were present or not in the training dataset to provide more insights into the model performance. The group subsets evaluated were: Grp1) both proteins and ligands not in training (Supplementary Figure 7), Grp2) proteins in training and ligands not in training (Supplementary Figure 8), and Grp3) ligands in training and proteins not in training (Supplementary Figure 9). Analysis on Grp1 and Grp3 show that the model is able to identify protein–ligand interactions for specific antibiotics not used in training but belonging to an antibiotic class used in training. This suggests protein interactions with new molecular derivatives of existing antibiotic classes could be identified. Importantly, both models exhibited higher precision, specificity, AUC, and PRAUC scores for Grp 3 versus Grp 1 or Grp 2, suggesting this approach can identify potential protein–ligand interaction for novel, unseen proteins (Supplementary Figure 10). Based on overall performance characteristics between the two models, the Random Forest classifier was chosen to characterize the novel AMR-associated protein for interactions with antibiotics.

**Figure 8:**
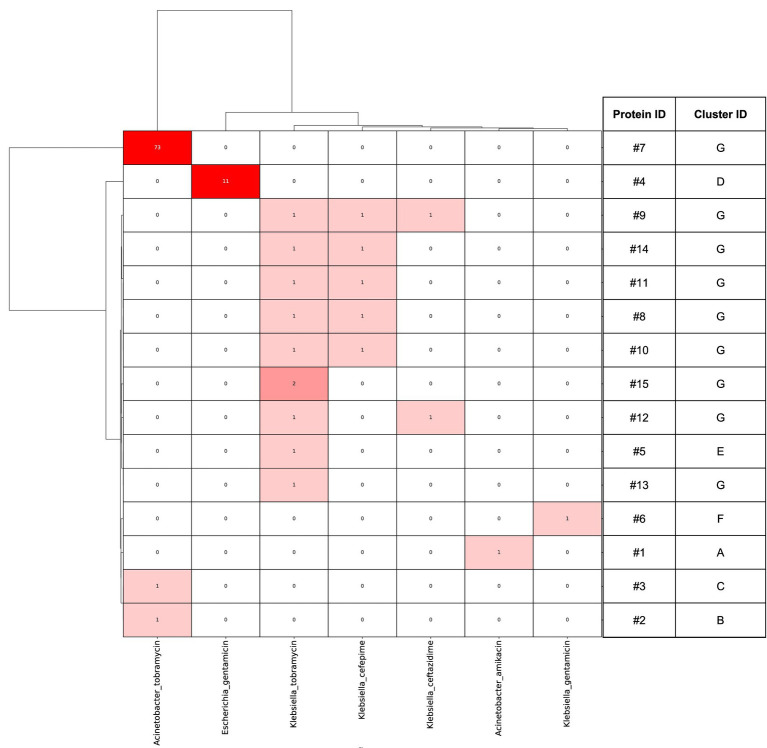
Protein-antibiotic interactions in novel AMR-associated genes were predicted using the random forest classifier model. Co-occurrence of pathogen-antibiotic combinations harboring proteins with identified ligand interactions was determined by hierarchical clustering based on Euclidean distance and shown as a clustermap. The number of genomes containing protein are indicated within cells of the clustermap. See Supplementary Table 2 for description of protein ID and cluster ID for each protein.

Using the random forest model, 45 protein-antibiotic combinations were identified with interaction probabilities >0.5. Of these, 15 proteins with amino acid length >100 residues were considered for further analysis because these may more reliably represent full-length instead of truncated annotations (Supplementary Table 2). Only protein-antibiotic interactions for aminoglycosides were identified in the hits. However, several protein sequences were also found in *Klebsiella* strains resistant to cefepime and ceftazidime (Figure 8). Interestingly, protein hits had a single or no protein domains annotated based on InterProScan analysis. The most common domain identified was “Domain of unknown function (DUF4158)” found in 8/15 of the hits. 3/15 proteins had no protein domains identified, suggesting uncharacterized structure and function. Of the 15 proteins identified, 9 belonged to the same cluster (“Cluster ID,” based on >30% sequence identify).

## Discussion

AMR pathogens remain a significant health crisis and require improved multi-modal approaches to identify and counteract these threats. Accordingly*, The National Action Plan for Combating Antibiotic-Resistant Bacteria* (CARB) issued by the U.S. Government emphasizes the necessity to mitigate the emergence and spread of AMR pathogens by advancing therapeutic and diagnostic technologies, including approaches for biosurveillance. Traditional biosurveillance for AMR pathogens have relied on laboratory-based methods to isolate infectious agents followed by antibiotic susceptibility testing or PCR-based assays for AMR genes against limited target panels. In the last decade, the increasing adoption of whole genome sequencing has enabled holistic characterization of AMR-associated determinants in pathogens. Thus, in the current study, a large-scale comparative genomic analysis of AMR pathogens was performed to identify key trends in resistance gene profiles and augment the biosurveillance landscape.

The BV-BRC’s repository of whole genome sequences paired with laboratory-based antibiogram data was utilized to investigate over 70,000 ESKAPE+ pathogen strains. The dataset included commonly used classes of antibiotics, thereby complementing the wide genomic diversity of the pathogen strains. By comparing protein-coding genes prevalence between susceptible versus resistant genome strains using a kmer-based GWAS approach, over 7,000 unique sequences were significantly enriched in resistant strains. When compared to CARD, 1,925 (29.9%) were homologous to known AMR genes, while over 5,000 (>70%) of enriched genes lacked sequence similarity. In addition to suggesting the presence of novel AMR-associated genes, this finding indicates that curated databases for AMR genes (Papp and Solymosi, 2022), such as CARD, may have gaps in their catalogs that could detrimentally impact the characterization of known or emerging threats.

Overall, the AMR gene profile for both known and novel-associated sequences was influenced according to pathogen-antibiotic combination. Pathogens with closer phylogeny and antibiotics with more similar molecular structures tended to have more comparable gene profiles. Additionally, only select known AMR genes predominated per pathogen-antibiotic combinations. Previous studies have described how pathogen resistomes converge on particular resistance genes/mechanisms in order to optimize survival against specific generations of beta-lactam or tetracycline antibiotics (Blake et al., 2025). When taken together, this suggests evolutionary pressures or niche-specific mechanisms may influence which AMR genes will prevail in pathogens. In support of this observation, horizonal gene transfer mechanisms were frequently identified as enriched AMR-associated genes, which may imply that selective exchange of AMR genes occur among more similar pathogen-antibiotic combination types. While horizonal gene transfer is well-described mechanism for AMR gene propagation, a recent study analyzing metagenomic sequencing data found that environmental niches may influence specific horizontal gene transfer mechanisms for AMR genes (Ebmeyer et al., 2025). Due to metadata limitation in the dataset, investigation into how environmental factors associated with AMR gene profiles was not possible. However, a potential hypothesis is that in addition to “classic” AMR genes, tracking and characterization of horizontal gene transfer mechanisms may improve the impact of biosurveillance efforts.

Previous reports have indicated that AMR can be driven by the co-production of multiple resistance genes simultaneously. For example, *Klebsiella* and other Gram-negative enteric pathogen have been identified that harbor two or beta-lactamase genes, especially carbapenemases which target imipenem, meropenem and other carbapenems (Bush and Bradford, 2020). Similarly, *Klebsiella* isolates co-harboring multiple aminoglycoside inactivation enzymes have been described (Huang et al., 2022). In the analysis, multi-gene resistance strains were identified across numerous antibiotic classes. However, based on statistical analysis by the chi-square tests, the co-presence of multiple for on-target antibiotic was infrequent. Aside from the vancomycin gene resistance cluster, enzymatic inactivation enzymes targeting aminoglycosides were found to be statistically co-harbored in the *Klebsiella*-tobramycin and *Salmonella*-streptomycin combinations. It is possible multi-gene resistance appears more frequently, but underrepresentation of these strains prevented statistical detection. Larger and more diverse datasets could enhance the profiling and identification of multi-gene resistance patterns in pathogens.

Within these analyses, insights were gained into the gene profiles for multidrug resistant mechanisms. 23/39 pathogen-antibiotic combination analyzed had strains with off-target AMR genes present, reinforcing the risk for multidrug resistance in pathogens. However, only 32 AMR gene pairs were identified that were significantly associated with multidrug resistant potential, with the most often occurring mechanisms being due to efflux pumps found in a diversity of pathogens. Interestingly, *Klebsiella* strains resistant to tobramycin often co-harbored inactivation enzymes for over 4 non-aminoglycoside antibiotic classes. In contrast, *Klebsiella* strains resistant to gentamicin (also an aminoglycoside) or *Escherichia* strains resistant to tobramycin did not share a diverse multidrug resistance profile. This observation suggests that different selection factors may contribute to the acquisition of multidrug resistant determinants and that these mechanisms could be specific for pathogen-antibiotic combinations.

Based on the employed comparative genomics approach, over 5,000 unique protein sequences without homology to known AMR gene in the CARD were identified. To investigate the potential function of these sequences, protein domain analysis was performed. As mentioned above, domains for horizonal gene transfer, such as plasmid and phage proteins, were frequently identified in the novel gene set. However, notable domains associated with antibiotic/natural product metabolism were also detected. Because most antibiotics are derived from biosynthetic gene clusters expressed by bacterial and fungal species, there are numerous examples of how these genes also contribute to resistance. For instance, the “producer hypothesis” proposes that a possible origin of antibiotic resistance is due to self-protecting mechanisms towards the cytotoxic molecule being produced by source organisms (Jiang et al., 2017; Wencewicz, 2019). These enzymes may have promiscuous substrate specificity or evolve new functions that confer resistance to other molecules, and this process may occur in tandem with genetic transmission to new hosts. In the biosynthesis of natural products, microbes frequently employ enzyme-mediate oxidation reactions (Wencewicz, 2019; Gibson et al., 2005; Tang et al., 2017). By co-opting similar oxidation mechanisms, microbes have acquired antibiotic resistance, such as FAD-dependent oxidoreductase enzymes inactivating tetracyclines (Gasparrini et al., 2020; Park et al., 2017). In the novel gene set, several protein domains associated with oxidation activity were identified, suggesting the possibility these proteins may potentiate resistance under similar mechanisms. Furthermore, several notable protein domains associated with antibiotic-associated metabolism/resistance were detected such as isochorismatase-like hydrolase, chloramphenicol acetyltransferase-like, glyoxalase/dihydroxybiphenyl dioxygenase, which may target to streptothricin, phenicol, and bleomycin-/fosfomycin-like molecules, respectively (Maruyama and Hamano, 2009). Likewise, known resistance gene may further mutate to accommodate non-canonical molecular scaffolds, as demonstrated by aminoglycoside N-acetyltransferase variants that can target and inactivate some fluoroquinolones (Vetting et al., 2008; Robicsek et al., 2006; Zubyk and Wright, 2021).

The analysis also detected several proteins with domains associated with known AMR mechanisms. These included multi-drug transporter, dihydrofolate reductase-like, topoisomerase, and pentapeptide-like repeat domains. While the identification of these proteins suggests that reference AMR gene database, like CARD, may have incomplete variants catalogs, additional verification is needed to confirm the role of these proteins with resistance phenotypes. Interestingly, 1,523 (29.1%) of novel genes associated with AMR identified in the current study lacked protein domains, which suggests the potential for novel mechanisms that may influence the genetic transmission of resistance mechanisms and/or potentiate antibiotic susceptibility.

Antibiotic inactivation enzymes are common mechanisms utilized to confer resistance and rely on direct interactions between protein and ligands. Thus, recent advances in AI/ML-based modeling of 3D protein structure with ligand docking could be an additional tool to identify and character AMR-associated proteins at high-throughput scales. Several previous studies have utilized structure–function modeling methods to investigate protein interactions with antibiotic targets, for both identifying potentially new antibiotic targets as well as identifying/classifying potentially new AMR-associated proteins (Yagimoto et al., 2024; Sunuwar and Azad, 2022; Wong et al., 2022). In this study, ESM-Fold and DiffDock2 was utilized to model protein structure and docking of antibiotic molecules, along with GNINA to refine docking poses and calculate binding affinity properties. To focus on the identification of antibiotic inactivation mechanisms, a random forest model was trained that also incorporated protein structure and molecular features along with binding affinity profiles based on known protein-antibiotic interactions for inactivation enzymes from the CARD. Using this random forest model, several novel protein sequences were identified that may engage with their target antibiotic molecules, including several proteins that lacked known domain regions. While further experimental work is needed to elucidate the contributions of these proteins to AMR phenotypes, this analysis approach provides a means to identify and characterize novel AMR-associated genetic determinants with statistical confidence. Furthermore, continued improvements in structure–function modeling tools along with expansions of curated AMR gene databases could enhance model performance and capabilities, such as the application of multi-classification algorithms to identify different resistance mechanisms simultaneously.

## Limitations

Despite the comprehensive analysis approach performed in this study, several limitations were noted. Firstly, it was observed that pathogen-antibiotic combinations included genomes that lacked AMR-associated genes. It is possible the analysis approach was underpowered to detect statistically associated genes contributed by these strains due their availability in the BV-BRC dataset. Similarity, because the BV-BRC dataset relies on open-source contributions of pathogen genomes and phenotypic metadata, there could be potential biases in the representation or distribution of strain lineages for each pathogen-antibiotic combination. To maximize overall statistical power per pathogen-antibiotic combination, this study did not subset the data by genomic subtypes or multi-drug resistance phenotypes, for example. Larger and more diverse datasets could be needed to address these limitations. Likewise, structure–function modeling of protein-antibiotic interactions using AI/ML approaches relies on robust training data. In this study, the CARD database provided training/testing data to enable the development of ML classifiers for prediction of protein-antibiotic interaction based on *in silico* binding, protein embedding, and molecular features. However, currently known AMR mechanisms are highly skewed to a subset of antibiotic classes, such as beta-lactams, which create challenges for developing generalized, highly performant prediction models. Increased protein–ligand interaction datasets, especially for AMR-related mechanisms, are needed to optimize structure–function modeling approaches that predict pathogenic phenotypes. Additionally, alternative mechanisms, such as epigenetic/transcriptional control of gene expression or post-translational modifications, may promote resistance instead of acquired AMR genes (Villalba de la Peña and Kronholm, 2024). Different data types, such as transcriptomics and proteomics, could be necessary to elucidate additional AMR mechanisms, especially for strains that lacked any detectable AMR-associated genes. Nonetheless, the analysis approaches demonstrated in this study provides a means to identify such pathogens so that cryptic mechanism of resistance can be further investigated.

## Conclusion

In conclusion, the findings support the study’s hypothesis that large-scale analysis of whole genome sequence data from pathogens followed by downstream structure–function predictions are feasible approaches to identify and characterize the genetic determinants of AMR in pathogens. Continued application of and investment in these computational tools may enhance biosurveillance efforts against AMR as well as promote new means to counteract emerging threats. In particular, larger and more diverse multi-omic datasets with associated AMR phenotyping are needed to optimize and validate the approaches and findings described in the current. By doing so, large-scale comparative genomics and structure–function modeling approaches could be translated into clinical applications such as improved diagnostics for AMR detection, prediction of treatment efficacy, and avenues to identify next-generation therapeutic modalities.

## Data Availability

The datasets presented in this study can be found in online repositories. The names of the repository/repositories and accession number(s) can be found in the article/supplementary material.
